# Development and Validation of a Nursing Students' Clinical Practice Stress Scale: A Mixed Methods Study

**DOI:** 10.1002/nop2.70424

**Published:** 2026-01-09

**Authors:** Anjali Chamika Rathnayaka Mudiyanselage, S. Samita

**Affiliations:** ^1^ Department of Nursing, Faculty of Allied Health Sciences University of Peradeniya Kandy Sri Lanka; ^2^ School of Nursing and Midwifery Griffith University Southport Australia; ^3^ Department of Crop Science, Faculty of Agriculture University of Peradeniya Kandy Sri Lanka

**Keywords:** clinical stressors, factor analysis, nursing students, reliability, stress, validity

## Abstract

**Background:**

In clinical environments, nursing students encounter a variety of stressors, which can significantly impact their well‐being, learning outcomes and the quality of care they provide to patients.

**Aims:**

To develop and validate the Nursing Students' Clinical Practice Stress Scale (NSCPSS) to measure clinical practice stressors in nursing students in Sri Lanka.

**Design:**

An exploratory sequential mixed methods design.

**Methods:**

The study was conducted in two phases. The NSCPSS items were developed in the qualitative phase based on data gathered through focus group interviews and a literature review. The quantitative phase focused on the psychometric evaluation of the scale, assessing its face, content, construct, convergent, discriminant validity and reliability using data from 183 nursing undergraduate students.

**Results:**

Four factors were extracted from 30 items through exploratory factor analysis: (1) lack of knowledge, skills and experience, (2) lack of academic communication and support systems, (3) challenges in managing academic and clinical demands and (4) challenges in the clinical learning environment. These four factors collectively explained 57.0% of the total variance. The confirmatory factor analysis demonstrated the acceptable goodness‐of‐fit indices. All factors showed reliability, with internal consistency and composite reliability indices > 0.6.

**Conclusion:**

The NSCPSS is a valid and reliable instrument to measure the clinical stressors experienced by undergraduate nursing students.

**Implications for Practice:**

The development and validation of the NSCPSS is a key step toward identifying stressors that undergraduate nursing students experience during clinical practice. It contributes to enhancing effective learning during clinical practice and students' well‐being and develops a resilient future nursing workforce capable of delivering high‐quality patient care.

**Reporting Method:**

Good Reporting of a Mixed Methods Study (GRAMMS) checklist.

**Patient or Public Contribution:**

No patient or public contribution.

## 1 Introduction

Nursing education consists of both theoretical and clinical components. Clinical training is considered the heart of nursing education and learning (Najafi et al. 2019). It allows nursing students to build competencies in applying knowledge, developing clinical and procedural skills and improving critical thinking and decision‐making abilities (Oermann and Shellenbarger 2020). Further, clinical training helps develop the professional identity and cultural competence of nursing students (Oermann and Shellenbarger 2020).

Clinical training can be stressful and emotionally challenging for nursing students (Stubin 2020). Nursing students experience stress in the clinical environment, ranging from mild to moderately high levels (Welch 2023). The most common sources of stress for nursing students include high patient care workload, increased academic workload with recurrent examinations and assignments, lack of knowledge and skills and problematic relationships with colleagues, clinical and academic staff (Harvey et al. 2024). Further, stressors can emerge due to a gap between the applicability of academic preparation to the clinical placement (Pulido‐Criollo et al. 2018).

Perceived stress can adversely impact the academic performance and clinical satisfaction of nursing students (Welch 2023). The level of stress experienced by the students is significantly associated with their overall health status (Mazalova et al. 2022). The greater perceived stress leads to more severe health problems (Wu et al. 2021). However, these detrimental effects can impede the preparation of competent nurses and ultimately affect the quality of patient care provided (Harvey et al. 2024). Therefore, nursing educators need to be aware of the factors that would increase stress among student nurses and help them identify appropriate coping strategies to overcome stressors.

Studies measuring and identifying nursing students' stress have used different instruments and methods (Welch 2023). Most researchers have used existing questionnaires, while others have undertaken qualitative studies to explore stress among nursing students. The scales, such as the perceived stress scale (PSS) (Cohen et al. 1983) and the student nurse stressor‐15 (SNS‐15) scale (Sheridan et al. 2019) have been widely used to assess stress among student nurses. However, these instruments may not fully capture the unique stressors experienced by Sri Lankan nursing students. For instance, the PSS measures the general perceived stress and was not specifically tailored to the clinical learning environment of nursing students. Similarly, the SNS‐15, although specific to nursing students, was developed in a different sociocultural and educational context, which may limit its relevance and validity in the Sri Lankan nursing education context.

In Sri Lanka, pre‐registration nursing education is delivered through the diploma programme and the university‐based Bachelor of Science (BSc) in Nursing programme (Al‐Worafi 2024). Although undergraduate nursing education within universities was introduced over two decades ago, student nurses enrolled in these programmes continue to encounter numerous challenges within their clinical learning environments. The factors, such as limited hospital resources and infrastructure, challenges in healthcare access, staffing shortages and culturally embedded norms surrounding communication and hierarchical authority within the healthcare setting, can all shape the stress experiences of Sri Lankan undergraduate nursing students in distinctive ways (Al‐Worafi 2024). The uniqueness of the sample needs to be considered when selecting a stress measure, and developing a new scale or modifying the existing scale can be necessary to fit the needs of some samples (Crosswell and Lockwood 2020). Therefore, a context‐specific tool is necessary to accurately assess and address clinical practice stress within the Sri Lankan undergraduate nursing student population. Thus, the aim of this study was to develop and validate the Nursing Students' Clinical Practice Stress Scale (NSCPSS) to measure the stressors of clinical practice in undergraduate nursing students in Sri Lanka.

## 2 Methodology

### 2.1 Study Design

The exploratory sequential mixed method study was conducted to develop and validate a scale to measure nursing students' clinical stressors. The qualitative data was collected and analysed first, and subsequently incorporated a quantitative phase based on the qualitative results (Creswell and Creswell 2023).

The qualitative phase, consisting of focus group discussions, was conducted first to explore the experiences of stressors in nursing students during clinical placements. The insights gained were used to generate an initial pool of scale items. The quantitative phase then followed, where the draft scale was tested for validity and reliability using exploratory and confirmatory factor analyses. The integration of qualitative and quantitative methods ensured that the scale was contextually relevant and psychometrically robust to measure the actual stressors of clinical practice in Sri Lankan undergraduate nursing students. Further details on how qualitative findings informed item generation are provided under the 2.3.4 data collection subsection of the quantitative phase.

The study was conducted in the Department of Nursing, Faculty of Allied Health Sciences, University of Peradeniya. This study was reported according to the Good Reporting of a Mixed Methods Study (GRAMMS) checklist (O'Cathain et al. 2008).

### 2.2 Qualitative Phase

#### 2.2.1 Design

A qualitative study was conducted using focus group discussions. The focus group method was an effective strategy for engaging participants during discussions and gathering relevant and unexpected insights, which contributed to creating new questionnaire items and refining existing ones (de Sousa et al. 2021).

#### 2.2.2 Participants, Sampling and Recruitment

The purposive sampling strategy was utilised to recruit participants for the qualitative phase of the study. The criterion for recruiting individuals for the focus group discussions was nursing undergraduates who successfully completed at least one clinical module. The maximum demographic variation in terms of gender and academic year was considered to capture diverse perspectives on the phenomenon being studied (Creswell and Poth 2018; Palinkas et al. 2015).

#### 2.2.3 Sample Size

The sample size was decided based on the principle of data sufficiency (LaDonna et al. 2021). This approach aligns with the evolving critical discussion of imprecise and inconsistent use of saturation in qualitative research (Braun and Clarke 2021). Data sufficiency focuses on the rigour of the analytical process and richness of data in meaningfully addressing the research questions (LaDonna et al. 2021), rather than saturation, which suggests a definitive point where no new codes or themes emerge (Braun and Clarke 2021). In this study, data sufficiency was reached after three focus groups with six students per group. A total of 18 students participated in the focus groups.

#### 2.2.4 Data Collection

Data were collected via an online (ZOOM) platform in the focus group discussions. The focus group discussion guide was developed based on the findings of the literature review on nursing students' stress in clinical practice. The sources for the literature review were identified by searching PubMed, ScienceDirect and Google Scholar. A nursing education expert reviewed the discussion guide before conducting the focus group discussions. Necessary adjustments were made based on the feedback to ensure clarity, relevance and accordance with the study aims.

Informed consent was obtained from participants for online focus groups. The confidentiality and anonymity of discussions were ensured. Each discussion was recorded and lasted up to 60 min. All interviews were transcribed verbatim, de‐identified and securely stored in a password‐protected computer file by the principal researcher.

#### 2.2.5 Data Analysis

Thematic analysis was used for data analysis in the qualitative phase. The thematic analysis involves the process of identifying, analysing and reporting patterns or themes within the data (Braun and Clarke 2006). In qualitative research, data analysis progresses concurrently with other study components, including data collection and report writing (Creswell and Creswell 2023). The analysis was guided by the six‐step framework outlined by Braun and Clarke (2019).

### 2.3 Quantitative Phase

#### 2.3.1 Design

A cross‐sectional quantitative study was conducted using a survey to address the aims of the study. A cross‐sectional design is appropriate for examining the prevalence of disease and traits, assessing attitudes and knowledge and conducting validation and reliability studies (Kesmodel 2018).

#### 2.3.2 Participants, Sampling and Recruitment

Participants of this study were undergraduate nursing students of the Faculty of Allied Health Sciences, University of Peradeniya. Participants must have completed at least one clinical module before the data collection period. A census sampling method was used to recruit participants for the quantitative phase of the study.

#### 2.3.3 Sample Size

There were four batches of students following the Bachelor of Science in Nursing degree programme in the Department of Nursing by the time of the data collection, and all of them had completed at least one clinical module. A total of 183 nursing students participated in the study. This sample size was deemed adequate based on power analysis for structural equation modelling using the web utility of computing power and minimum sample size for root mean square error of approximation (RMSEA) (Preacher and Coffman 2006). The degrees of freedom for the model with 30 items and six latent variables was 414, calculated by subtracting the total number of estimated parameters (81) from the total number of observations (495) (Myers et al. 2016). The other required parameters for sample size calculation were type I error (*α*) (0.05), desired power (0.80) and RMSEA (null = 0.05 and alternative = 0.01) (Myers et al. 2016). The minimum sample size required for this study was 81. The final sample of 183 exceeded this threshold, supporting the adequacy of the sample size for factor analysis.

#### 2.3.4 Data Collection

Data were collected between September 2022 and January 2023 using a self‐administered questionnaire consisting of demographic information, including age, gender, year of study, family income, living status during clinical, satisfaction regarding studying nursing, and intention to change profession in the future and 30 potential NSCPSS items. Representatives from each batch were asked to distribute and collect all completed surveys and hand them over to the principal researcher.

##### 2.3.4.1 Item Generation for the NSCPSS

A total of 263 codes were extracted by analysing the qualitative data gathered from focus group discussions. The identified codes were grouped into 36 sub‐themes. These sub‐themes were considered as the initial items for the NSCPSS. Sub‐themes were categorised into six themes, including stressors from the clinical learning environment, stressors from barriers to daily life and relationships, stressors from lack of knowledge and skills, stressors from caring for patients, stressors from the academic staff and stressors from the nature of the academic programme. Emerged themes were considered as the latent variables for the subsequent analysis.

#### 2.3.5 Data Analysis

The face, content and construct validity, internal consistency and composite reliability of the NSCPSS were evaluated in the quantitative phase. Data were analysed using SPSS Statistics 30.0 and AMOS 30.0 (IBM Corp., Armonk, NY, USA).

##### 2.3.5.1 Face Validity

The list of scale items was presented to three recent nursing graduates from the Department of Nursing, Faculty of Allied Health Sciences, University of Peradeniya. They commented on the appropriateness, necessity, clarity and comprehensiveness of each item. Based on the comments, the wordings of some items were revised.

##### 2.3.5.2 Content Validity

Content validity was assessed using both qualitative and quantitative methods. For a qualitative content validity evaluation, 13 experts in nursing education, psychiatry and psychology were asked to comment on the wording, item allocation and scoring of the NSCPSS items. Based on their comments, the scale items were revised.

The validity of the instrument was evaluated using the content validity ratio (CVR) and content validity index (CVI) (Lawshe 1975; Polit and Beck 2006). Accordingly, 13 experts were asked to rate the necessity of each NSCPSS item on a 3‐point scale: (1) Necessary; (2) useful but not necessary; or (3) not necessary. The CVR was calculated based on the following formula:

$$\text{CVR}=\frac{n_{E}-N/2}{N/2}$$

where $n_{E}$ is the experts who selected the necessary option and N is the total number of experts.

The items with CVR values lower than 0.49 were removed according to the Lawshe table (Lawshe 1975).

For the CVI calculation, the same experts were asked to rate the relevancy of each item to undergraduate nursing students’ stressors in a clinical setting on a 4‐point scale: (1) not relevant; (2) somewhat relevant; (3) quite relevant; and (4) highly relevant. The item‐level CVI (I‐CVI) was computed by dividing the number of experts who rated the item as ‘3’ or ‘4’ by the total number of experts. The I‐CVI with values > 0.7 was considered acceptable. Moreover, an average scale‐level CVI (S‐CVI/Ave) was evaluated by averaging the I‐CVI scores. An S‐CVI/Ave of > 0.9 was considered acceptable (Polit and Beck 2018).

##### 2.3.5.3 Construct Validity

Construct validity was evaluated using exploratory factor analysis (EFA) and confirmatory factor analysis (CFA). The dataset was randomly divided into two subsets using SPSS to enable EFA to be conducted on the first subset (95 participants) and CFA performed on the second subset (88 participants). The Kaiser‐Meyer‐Olkin (KMO) and Bartlett tests were conducted to assess the sampling adequacy for the EFA. The KMO value > 0.70, and the statistically significant Bartlett test value (*p* < 0.05) were considered acceptable (Watkins 2018). The latent factors of the NSCPSS were extracted via principal axis factoring (PAF) with Promax rotation. The Kaiser criterion (eigenvalues > 1), visual scree plot analysis and parallel analysis were applied as factor retention approaches. The primary factor loading cutoff was considered ≥ 0.40, and the cross‐loading cutoffs ≥ 0.02 as the constructs are broad, multidimensional and elusive (Howard 2023).

A CFA was conducted to confirm the extracted factor model determined by EFA. The maximum likelihood estimation (MLE) robust extraction method was used to estimate the parameter estimation. To assess the overall quality of model fit, the following model fit indices were considered:

- Chi‐square test (*χ* ^2^): The *χ* ^2^ test with *p* > 0.05 would be ideal for optimal model fitting. The ratio of chi‐square and degrees of freedom was also assessed. If the ratio is ≤ 2, the fit is superior; < 5, the fit is good; and > 5, the fit is unacceptable (Chang et al. 2019). However, decisions need to be made with caution as the chi‐square test is sensitive to sample size (Abell et al. 2009; Fan et al. 2016).
- Root mean square error of approximation (RMSEA): RMSEA measures model fit based on the degree of non‐centrality of the chi‐square statistic (Abell et al. 2009). Values closer to zero indicate a better model fit, and higher values indicate a lack of fit. Recommended values under 0.06 indicate a perfect fit (Fan et al. 2016).
- Comparative fit index (CFI): CFI evaluates the proportion of variance explained in the covariance matrix. Values range between 0 and 1, with higher values indicating perfect model fit. The CFI should be close to 0.95 or higher in practice (Abell et al. 2009; Fan et al. 2016).
- Tucker‐Lewis index (TLI): TLI is a non‐normed fit index ranging from 0 to 1. A higher value indicates more improvement from the null model, and a TLI > 0.90 is considered acceptable (Abell et al. 2009; Fan et al. 2016).

##### 2.3.5.4 Convergent and Discriminant Validity

Convergent validity ensures that items intended to measure the same construct are highly correlated. It was measured using the average variance extracted (AVE) and composite reliability (CR). An AVE of 0.5 or higher suggests good convergent validity (Hair et al. 2020) and a minimum accepted value of 0.60 is needed to achieve high CR (Purwanto and Sudargini 2021). However, the study should be continued if the AVE < 0.5 and the CR is larger than 0.60 (Lam 2012).

Discriminant validity ensures that constructs that are theoretically distinct are not highly correlated. It was assessed by the Fornell‐Larcker criterion (Fornell and Larcker 1981) and Heterotrait‐monotrait (HTMT) criterion (Henseler et al. 2015). The Fornell‐Larcker criterion was evaluated by comparing the square root of each AVE in the diagonal with the correlation coefficients (off‐diagonal) for each construct in the relevant rows and columns (Fornell and Larcker 1981). HTMT values below 0.85 (or 0.90) suggest acceptable discriminant validity (Henseler et al. 2015).

##### 2.3.5.5 Reliability

The reliability of a questionnaire indicates the consistency of the survey results (Tsang et al. 2017). Internal consistency was estimated using Cronbach's alpha. Cronbach's alpha of 0.70 was considered adequate internal consistency (Tsang et al. 2017).

##### 2.3.5.6 Scoring

The NSCPSS items were scored on a linear 5‐point Likert scale: 1 (never) to 5 (very often).

### 2.4 Ethical Considerations

Before conducting the study, ethical approval was obtained from the Ethics Review Committee, Faculty of Allied Health Sciences, University of Peradeniya, Sri Lanka (AHS/ERC/2021/111). The researcher ensured that participation was voluntary and that the subjects' confidentiality, anonymity, privacy and safety were maintained throughout the study. Limited demographic information was collected to protect anonymity, and a third person was used to collect survey data to minimise coercion.

## 3 Results

### 3.1 Demographic Characteristics of Participants

Table 1 presents the demographic characteristics of the overall sample and the subsamples of EFA and CFA with frequencies and percentages. A total of 183 undergraduate nursing students participated in this study. The students' mean age was 24.68 (SD = 1.47), and most were females (*n* = 135, 73.8%). Most participants had adequate family income (*n* = 112, 61.2%) and were living in university accommodations (hostels) with colleagues during the clinical period (*n* = 177, 96.7%). Regarding the level of satisfaction in studying nursing, 62.3% (*n* = 114) indicated that they were satisfied and 50.8% (*n* = 93) of participants mentioned their intention to change their profession from nursing to any other profession after graduation.

**TABLE 1:** Demographic characteristics of the overall sample and subsamples.

Sample	Gender		Age	Year of study			
	Male	Female	Mean ± SD	First	Second	Third	Fourth
Total (*N* = 183)	48 (26.2)	135 (73.8)	24.68 ± 1.47	47 (25.7)	44 (24.0)	42 (23.0)	50 (27.3)
EFA (*N* = 95)	22 (23.2)	73 (76.8)	24.56 ± 1.53	28 (29.5)	23 (24.2)	16 (16.8)	28 (29.5)
CFA (*N* = 88)	26 (29.5)	62 (70.5)	24.82 ± 1.39	19 (21.6)	21 (23.9)	26 (29.5)	22 (25.0)

### 3.2 Face and Content Validity

Five items were revised, and two items were separated, as they were perceived to represent two distinct concepts during the face validity process. After face validity evaluation, 38 items were grouped into stressors related to the following six categories: the clinical learning environment (9 items), barriers to daily life and relationships (5 items), lack of knowledge and skills (4 items), caring for patients (4 items), academic staff (4 items) and the nature of the academic programme (12 items).

Eight items with CVR values < 0.49 were removed through content validity evaluation by 13 experts. The I‐CVI values for the remaining 30 items were ≥ 0.7, and the value of S‐CVI/Ave for the 30‐item NSCPSS was 0.89.

### 3.3 Construct Validity

**FIGURE 1:**
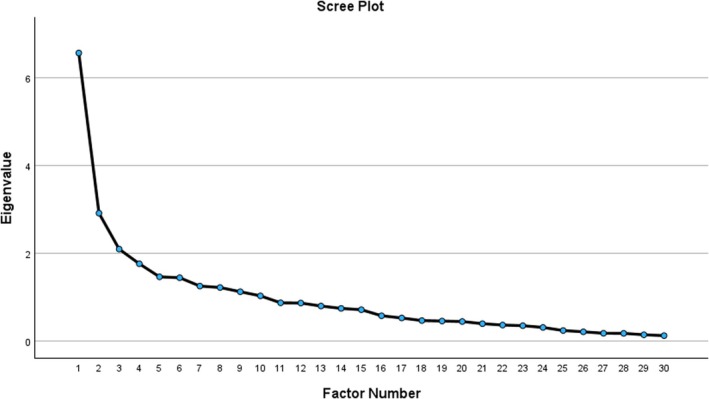
Scree plot of the selected 30‐item Nursing Students' Clinical Practice Stress Scale (NSCPSS).

An EFA was performed on the data obtained from 95 nursing undergraduates. The Keiser‐Meyer‐Olkin test value was 0.76, and Bartlett's test value was 654.26 (*p* < 0.001), indicating sample adequacy for EFA. Based on the Kaiser criterion (eigenvalues > 1), visual scree plot analysis and parallel analysis, four main factors were extracted: Lack of knowledge, skills and experience (5 items), lack of academic communication and support systems (4 items), challenges in managing academic and clinical demands (6 items) and challenges in the clinical learning environment (4 items). Figure 1 presents the scree plot before item deletion.

One item (Q26) was deleted from the initial EFA analysis due to cross‐loading and the difference between loadings < 0.02. Another 10 items (Q2, Q3, Q4, Q5, Q6, Q10, Q12, Q18, Q19, Q25) were deleted based on the criteria of factor loadings < 0.40. A total of 19 items across four factors remained after EFA analysis. The eigenvalues of the extracted four factors were 4.90, 2.68, 1.80 and 1.45, respectively, and they explained 57.0% of the total variance of the NSCPSS (Table 2).

**TABLE 2:** Factors extracted from the Nursing Students' Clinical Practice Stress Scale (NSCPSS).

Factors	Items	Factor loading	*h* ^2^	*λ*	Variance (%)
Lack of knowledge, skills and experience					
q13	Lack of knowledge of diseases and treatments	0.609	0.560	4.898	25.78
q14	Lack of experience in providing patient care	0.829	0.651		
q15	Lack of skills in providing patient care	0.939	0.719		
q16	Lack of skills in handling medical equipment	0.805	0.547		
q17	Challenges of caring for critically ill patients	0.558	0.468		
Lack of academic communication and support systems					
q21	Lack of support and guidance from the academic staff of the university	0.646	0.451	2.676	14.08
q22	Academic staff do not fairly evaluate students' performances	0.851	0.649		
q23	Lack of teaching, practical and time management skills of academic staff	0.773	0.567		
q29	Insufficient explanation of the module introduction and objectives	0.400	0.432		
Challenges in managing academic and clinical demands					
q1	Inflexible continuous clinical placements	0.442	0.159	1.801	9.48
q11	Issues created by financial difficulties	0.445	0.220		
q24	Requirement of completion of 100% compulsory attendance for clinical practice	0.559	0.239		
q27	Not having a common procedure manual for undergraduate nursing students	0.454	0.469		
q28	Pressure from higher academic workload	0.532	0.452		
q30	Strict deadlines for assignment/case study submission	0.442	0.331		
Challenges in the clinical learning environment					
q7	Lack of facilities for dining and resting at the hospital	0.517	0.291	1.454	7.65
q8	Difficult to apply learned procedure steps in university to clinical setting	0.605	0.446		
q9	Lack of resources to practice the learned procedures in the wards	0.833	0.685		
q20	Lack of formative evaluations for clinical training	0.478	0.463		

*Note:* *h* ^2^: communality, *λ*: eigenvalue.

The derived factor structure was evaluated through CFA using data collected from 88 nursing undergraduates. In total, 19 items were entered into the CFA. After the initial model run, modification indices suggested correlations between measurement errors, including items 13 and 16, 15 and 23, 16 and 17, 29 and 30, and 11 and 28. In this model, most items have factor loadings > 0.50, with corresponding latent variables, except for two items (Q1 and Q7).

The two items (Q1 and Q7) were removed in the subsequent step, and the model was reanalysed to assess its revised structure. Q1 (inflexible continuous clinical placements) and Q7 (lack of facilities for dining and resting at the hospital) demonstrated standardised factor loadings of 0.36 and 0.47, respectively. These values were less than the recommended standardised factor loading of 0.5 for construct validity in CFA (Hair et al. 2018). In addition, Q1 overlapped conceptually with Q24 (requirement of completion of 100% compulsory attendance for clinical practice), which more directly captured the perceived stress related to rigid attendance requirements. Further, Q7 was more infrastructural and peripheral to the psychological aspects of clinical stressors, emphasised in the final model. The removal of Q1 and Q7 improved both model fit and conceptual clarity.

**FIGURE 2:**
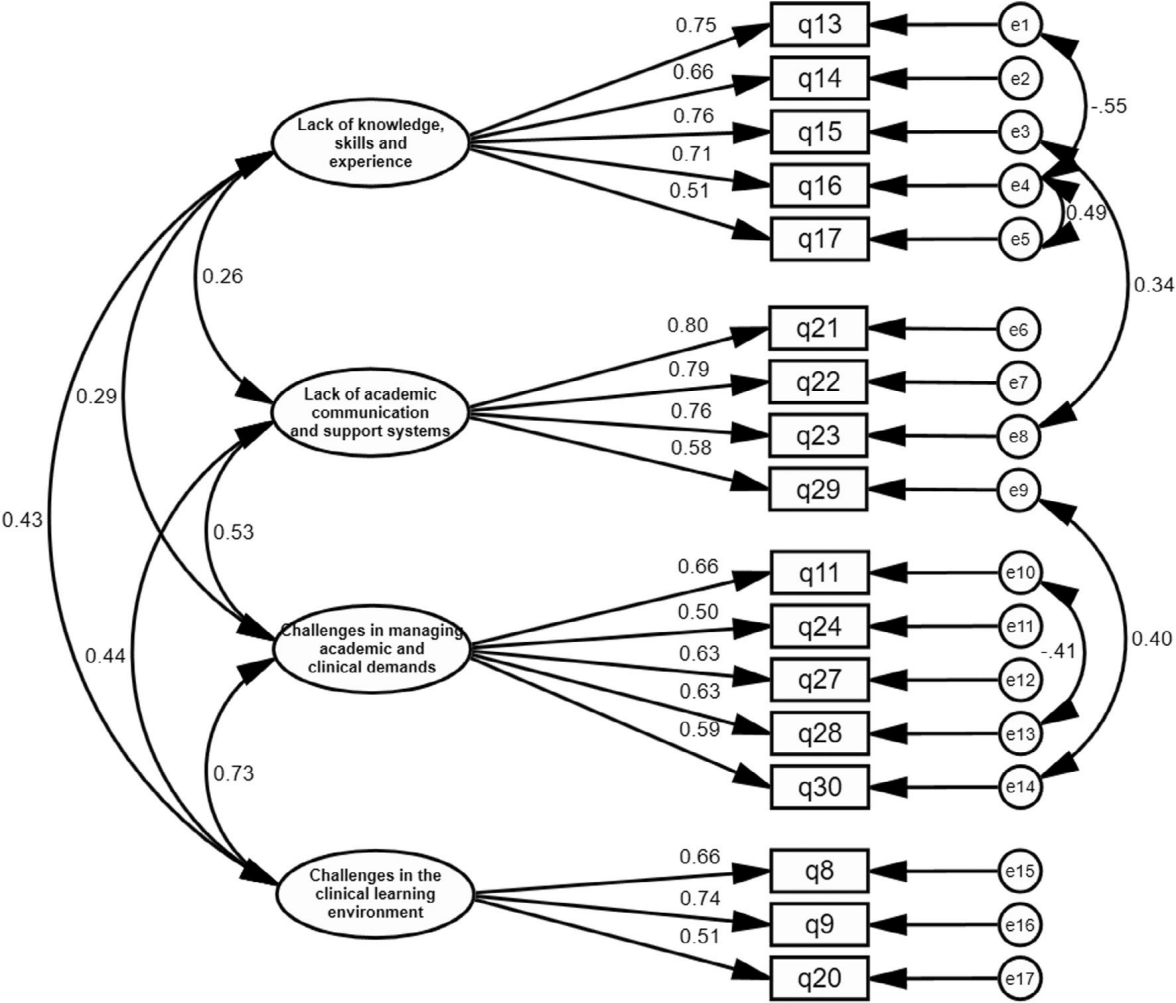
The confirmatory factor analysis model of the Nursing Students' Clinical Practice Stress Scale (NSCPSS).

The final model, shown in Figure 2, reveals that all factor loading values were > 0.5. The chi‐square index for the goodness‐of‐fit was 130.93 (*p* = 0.066) after the model correction. The ratio of chi‐square and degrees of freedom was 1.21, which suggested the superior fit. The other calculated model fit indices were RMSEA = 0.049, CFI = 0.954 and TLI = 0.942. These indicators confirmed the goodness‐of‐fit of the model.

### 3.4 Convergent and Discriminant Validity

The CR for all constructs of NSCPSS were > 0.60, and therefore, convergent validity can be accepted. Table S1 summarises the CR, AVE and the correlation coefficients between the constructs. The Fornell‐Larcker criterion of the square root of each AVE higher than the correlation coefficients for each construct was achieved except for the ACD‐CLE construct. Therefore, HTMT analysis was also conducted to confirm the discriminant validity. Table S2 depicts the output from the HTMT analysis. All calculated HTMT values were below 0.85, confirming the discriminant validity of the NSCPSS.

### 3.5 Reliability

Cronbach's *α* was calculated for each subscale of the NSCPSS to assess internal consistency reliability. The *α* coefficient for the lack of knowledge, skills and experience; lack of academic communication and support systems; challenges in managing academic and clinical demands; and challenges in the clinical learning environment subscales was 0.83, 0.79, 0.69 and 0.69, respectively.

### 3.6 Scoring

The 17 items of NSCPSS were scored on a 5‐point Likert scale. The total score of the scale ranges from 17 to 85. Higher scores indicate a higher level of clinical practice stress in nursing undergraduates.

## 4 Discussion

This study aimed to develop and validate a scale (NSCPSS) to measure the stressors of clinical practice in nursing students. The findings of the study suggested that the scale is valid and reliable. The NSCPSS contains 17 items grouped in four subscales: lack of knowledge, skills and experience; lack of academic communication and support systems; challenges in managing academic and clinical demands; and challenges in the clinical learning environment. The acceptable explained variance of the scale confirms its ability to measure perceived stress among nursing undergraduate students in clinical environments.

The first subscale identified was lack of knowledge, skills and experience. This subscale explained the greatest proportion of variance among all identified subscales. Similarly, other studies have reported that insufficient clinical knowledge and skills were among the most prevalent sources of stress for nursing students (Najafi et al. 2015; Rafati et al. 2021). Updated knowledge and skills are essential for nurses to provide quality patient care (Gassas 2021). Therefore, students are afraid that their insufficient knowledge and skills may lead to harm or injury to patients (Dias et al. 2024). Further, students perceived limited knowledge as a significant source of stress, as it often includes unfamiliarity with medical terminology and clinical procedures (McCarthy et al. 2018). Lack of experience in providing care and making decisions has also been a common stressor for nursing students (Wu et al. 2021). These stressors may negatively affect students' confidence in providing care to patients in healthcare settings (Dias et al. 2024).

The lack of academic communication and support systems was the second subscale. This refers to systematic deficiencies in the academic environment and staff, leading to undergraduate nursing students' stress. Previously, ineffective educators have been highlighted as a significant concern in a study evaluating nursing students' clinical experiences (Rafati et al. 2021). Najafi et al. (2019) reported a lack of efficiency, clinical skills and improper student evaluations by the academic and clinical staff. In addition, student evaluation by academic staff was a significant factor influencing the anxiety levels of nursing students during clinical placements (Mazalova et al. 2022). Ignoring the needs of the students and lack of support from faculty or university may lead to emotional trauma and psychological effects, which eventually result in a lack of interest and unfavourable attitudes toward the nursing profession, learning difficulties, anxiety and stress (Najafi et al. 2015). Assigning academic and clinical staff with adequate knowledge and skills can alleviate unnecessary stress among students and enhance the overall learning environment (Najafi et al. 2015).

The challenges in managing academic and clinical demands were the third subscale. McCarthy et al. (2018) reported that the academic environment, examinations and assignments were associated with academic stressors among nursing undergraduates. They also highlighted that the number of examinations and assignments, and meeting the deadlines for assignments, were perceived as stressful for students. Higher academic workloads among nursing undergraduates were reported in several studies (Mazalova et al. 2022; Sanchez de Miguel et al. 2020; Sheridan et al. 2019; Wu et al. 2021). In addition, financial stressors, including not being eligible for grants or scholarships, unavailability of part‐time work and the high cost of living outside the home, have also been identified as a source of stress for nursing undergraduates (McCarthy et al. 2018).

The fourth subscale was the challenges in the clinical learning environment. McCarthy et al. (2018) found that the stress from the clinical environment was more prominent than that from academic or financial sources. Mazalova et al. (2022) reported that the clinical environment was a significant predictor of academic stress. Further, the lack of facilities and unavailability of necessary equipment resulted in wasted time and delays in patient care, creating an educational environment that students found unpleasant (Najafi et al. 2019). Sheridan et al. (2019) reported that a clinical placement lacking resources may reduce students' intention to work in the same profession after graduation.

In reviewing the relationships among the four subscales, the first two subscales demonstrated distinct conceptual boundaries. However, a degree of conceptual and statistical overlap was observed between the final two subscales: challenges in managing academic and clinical demands (ACD) and challenges in the clinical learning environment (CLE). Specifically, the ACD‐CLE construct did not meet the Fornell‐Larcker criterion (Fornell and Larcker 1981); however, HTMT values remained within acceptable limits, indicating that discriminant validity was still supported (Henseler et al. 2015). This overlap may reflect the practical realities of nursing education in the Sri Lankan context, where academic and clinical demands often emerge from the same structural constraints. For instance, the struggles of nursing students with high academic workloads, compulsory attendance and lack of procedural clarity (as captured in ACD) are frequently intensified by limited clinical resources, difficulty applying theory to practice and inadequate formative feedback (as reflected in CLE). This interdependence may explain the statistical proximity observed in the model and suggests that future refinement of the scale could explore certain items that should be reassigned or reworded to isolate the constructs better.

The NSCPSS was developed specifically to reflect the cultural and educational context of Sri Lankan undergraduate nursing students. However, many of the stressors it measures, such as limited clinical resources, high academic workload, challenging clinical learning environment and insufficient clinical supervision, are common across nursing programmes in low‐ and middle‐income countries (LMICs) (Shaban et al. 2012; Toqan et al. 2023). Nursing education in LMICs is often challenged by shortages in nursing academics, scarcity of resources and inconsistency between curricula and local healthcare needs (Sommers and Rio 2020). These shared structural and contextual factors suggest the possibility of NSCPSS adaptation in similar educational settings. Future research is needed to explore the cross‐cultural relevance of NSCPSS and validation in other LMICs, in order to broaden its applicability and contribute to a more global understanding of clinical practice stress in nursing education.

### 4.1 Strengths and Limitations

Several strengths and limitations can be identified in the NSCPSS and its development process. The development of NSCPSS was based on empirical data and a comprehensive literature review. The construct validity of the NSCPSS was evaluated using both exploratory and confirmatory factor analyses. One limitation of the study was that the study was limited to one institution in Sri Lanka. However, the selected institution was one of the five state universities conducting pre‐registration BSc Nursing degree programmes in Sri Lanka.

### 4.2 Implications for Practice

The development and validation of the Nursing Students' Clinical Practice Stress Scale (NSCPSS) is a significant step in identifying stressors that undergraduate nursing students experience during their clinical practice. The NSCPSS enables the early identification of vulnerable students, emphasising the implementation of targeted interventions to minimise the adverse effects of clinical practice stress. Further, it helps educators to understand specific challenges that students face in an actual clinical environment, prepare them for dealing with those challenges and facilitate a more supportive educational environment. These measures are important for enhancing effective learning and the well‐being of nursing students during clinical practice. In addition, they support developing a resilient future nursing workforce capable of delivering high‐quality patient care.

## 5 Conclusion

The NSCPSS is a valid and reliable instrument that can be used to measure the stressors experienced by undergraduate nursing students during clinical practice. It has the potential to inform the strategies to reduce stress and enhance students' clinical experience. Further research is needed to adapt and validate the NSCPSS for use in diverse cultural and clinical settings, ensuring its broader applicability and effectiveness.

## Author Contributions

A.C.R.M.: conceptualisation, methodology, data curation, formal analysis and writing – original draft. S.S.: conceptualisation, methodology, writing – review and editing and supervision.

## Funding

The authors have nothing to report.

## Conflicts of Interest

The authors declare no conflicts of interest.

## Supporting information


**Data S1:** nop270424‐sup‐0001‐AppendixS1.docx.

## Data Availability

The data that support the findings of this study are available from the corresponding author upon reasonable request.

## Good Reporting of a Mixed Methods Study (GRAMMS) Checklist

**Table:**

Guideline	Page no.
1. Describe the justification for using a mixed methods approach to the research question	5
2. Describe the design in terms of the purpose, priority and sequence of methods	5
3. Describe each method in terms of sampling, data collection and analysis	6–13
4. Describe where integration has occurred, how it has occurred and who has participated in it	5 & 9
5. Describe any limitation of one method associated with the present of the other method	20
6. Describe any insights gained from mixing or integrating methods	17–21
