# Supplementary material for: Development and Validation of a Nursing Students' Clinical Practice Stress Scale: A Mixed Methods Study
Source: Nurs Open. 2026 Jan 9;13(1):e70424. doi: 10.1002/nop2.70424 (PMC12789653; doi:10.1002/nop2.70424)
Supplement: Supplementary file 1 — Data S1: nop270424‐sup‐0001‐AppendixS1.docx. [file NOP2-13-e70424-s001.docx]

Supplementary File 1

Table S1: Composite reliability (CR), the square root of the average variance extracted (AVE) (in bold) and correlations between constructs (off-diagonal)

| **Latent constructs** | **CR** | **AVE** | **Latent constructs** | | | |
| --- | --- | --- | --- | --- | --- | --- |
|  |  |  | **KSE** | **ACS** | **ACD** | **CLE** |
| Lack of knowledge, skills and experience (KSE) | 0.811 | 0.466 | **0.683** |  |  |  |
| Lack of academic communication and support systems (ACS) | 0.826 | 0.547 | 0.259 | **0.739** |  |  |
| Challenges in managing academic and clinical demands (ACD) | 0.740 | 0.364 | 0.287 | 0.533 | **0.603** |  |
| Challenges in the clinical learning environment (CLE) | 0.677 | 0.417 | 0.426 | 0.440 | 0.725 | **0.645** |

Supplementary File 2

Table S2: Heterotrait-monotrait (HTMT) criterion results

|  | **KSE** | **ACS** | **ACD** | **CLE** |
| --- | --- | --- | --- | --- |
| **KSE** | - |  |  |  |
| **ACS** | 0.37 | - |  |  |
| **ACD** | 0.24 | 0.58 | - |  |
| **CLE** | 0.52 | 0.52 | 0.56 | - |

KSE: Lack of knowledge, skills and experience; ACS: Lack of academic communication and support systems; ACD: Challenges in managing academic and clinical demands; CLE: Challenges in the clinical learning environment
